# 2-Carb­oxy-6-(quinolin-1-ium-8-yl­oxy)benzoate

**DOI:** 10.1107/S1600536812013980

**Published:** 2012-04-13

**Authors:** Li-Mao Cai, Xin Fang, Mei-Jin Lin, Jin Xie, Jun-Dong Wang

**Affiliations:** a Department of Chemistry, University of Fuzhou, Fuzhou 350108, People’s Republic of China

## Abstract

In the zwitterionic title compound, C_17_H_11_NO_5_, the dihedral angle between the two aromatic rings is 76.90 (7)°. The dihedral angles between the carboxyl groups and the benzene ring are 64.02 (9) and 21.67 (9)°, the larger angle being associated with an intra­molecular N—H⋯O_carbox­yl_ hydrogen bond, resulting from proton transfer from the carb­oxy­lic acid group to the quinoline N atom and giving an *S*(9) ring motif. In the crystal, mol­ecules are connected by O—H⋯O hydrogen bonds into chains extending along the *b*-axis direction. An overall two-dimensional network structure is formed through π–π inter­actions between the quinoline rings [minimum ring-centroid separation = 3.6068 (6) Å].

## Related literature

For the use of phthalic acid derivatives in the construction of coordination polymers, see: Su *et al.* (2007 ▶); Zhang, Su, Li *et al.* (2006) ▶. For their potential applications, see: Wang *et al.* (2009 ▶); Zhang, Su, Song *et al.* (2006) ▶. For graph-set analysis, see: Etter *et al.* (1990 ▶).
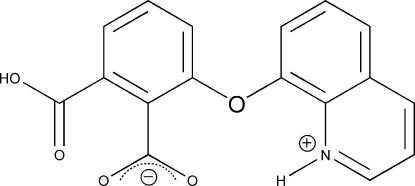


## Experimental

### 

#### Crystal data

C_17_H_11_NO_5_
*M* *_r_* = 309.27
Monoclinic, 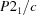

*a* = 7.7337 (15) Å
*b* = 11.580 (2) Å
*c* = 15.260 (3) Å
β = 98.43 (3)°
*V* = 1351.9 (5) Å^3^
*Z* = 4
Mo *K*α radiation
μ = 0.11 mm^−1^
*T* = 293 K
0.47 × 0.45 × 0.10 mm

#### Data collection

Rigaku Saturn 724 CCD area-detector diffractometer
Absorption correction: numerical (*NUMABS*; Higashi, 2000 ▶). *T*_min_ = 0.948, *T*_max_ = 0.989
11030 measured reflections
3079 independent reflections
2899 reflections with *I* > 2σ(*I*)
*R*_int_ = 0.041

#### Refinement

*R*[*F* ^2^ > 2σ(*F* ^2^)] = 0.058
*wR*(*F* ^2^) = 0.157
*S* = 1.27
3079 reflections
216 parameters
1 restraint
H atoms treated by a mixture of independent and constrained refinement
Δρ_max_ = 0.30 e Å^−3^
Δρ_min_ = −0.29 e Å^−3^

### 

Data collection: *CrystalClear* (Rigaku, 2007 ▶); cell refinement: *CrystalClear*; data reduction: *CrystalClear*; program(s) used to solve structure: *SHELXS97* (Sheldrick, 2008 ▶); program(s) used to refine structure: *SHELXL97* (Sheldrick, 2008 ▶); molecular graphics: *PLATON* (Spek, 2009 ▶); software used to prepare material for publication: *SHELXL97*.

## Supplementary Material

Crystal structure: contains datablock(s) I, global. DOI: 10.1107/S1600536812013980/zs2185sup1.cif

Structure factors: contains datablock(s) I. DOI: 10.1107/S1600536812013980/zs2185Isup2.hkl

Supplementary material file. DOI: 10.1107/S1600536812013980/zs2185Isup3.cml

Additional supplementary materials:  crystallographic information; 3D view; checkCIF report

## Figures and Tables

**Table 1:** Hydrogen-bond geometry (Å, °)

*D*—H⋯*A*	*D*—H	H⋯*A*	*D*⋯*A*	*D*—H⋯*A*
O2—H2*A*⋯O4^i^	0.93 (4)	1.67 (4)	2.584 (2)	165 (4)
N1—H1*A*⋯O5	0.90 (2)	1.67 (2)	2.570 (2)	179 (5)

Symmetry code: (i) 
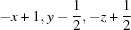
.

**Fig. 1.**
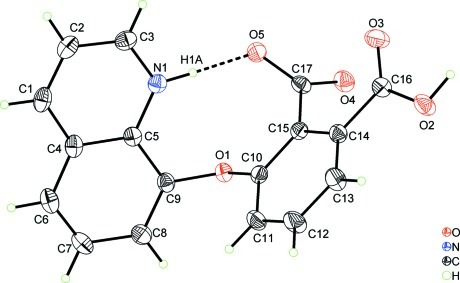
The molecular structure of the title compound, showing the atom-labelling scheme, with the intramolecular hydrogen bond shown as a dashed line. Displacement ellipsoids are drawn at the 30% probability level.

**Fig. 2.**
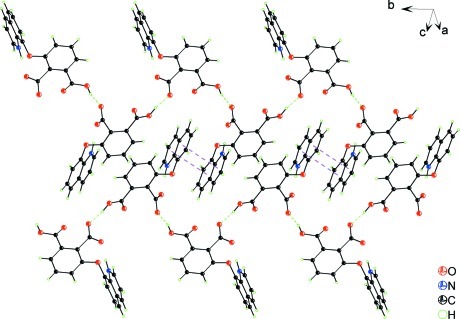
The two-dimensional supramolecular structure formed through hydrogen-bonds and π–π stacking interactions. Hydrogen bonds are shown with green dashed lines and π–π stacking interactions are shown by blue dashed lines.

## supplementary crystallographic information

### Comment

Phthalic acid derivatives have proved useful as ligands for the construction of coordination polymers which have a number of potential applications (Zhang, Su, Song *et al.*, 2006; Zhang, Su, Li *et al.*, 2006; Su *et al.*, 2007; Wang *et al.*, 2009). As a part of our investigation of the rare earth coordination networks based on these phthalic acid derivatives, we report here the crystal structure of the title compound, the zwitterionic substituted phthalic acid C_17_H_11_NO_5_ (Fig. 1). In this molecule, the carboxylic acid substituent group at C15 has protonated the quinoline N-atom, giving an intramolecular N—H···O_carboxyl_ hydrogen-bonding association [graph set *S*9 (Etter *et al.*, 1990)]. The dihedral angles between the carboxyl groups and the benzene ring are 64.02 (9)° and 21.67 (9)°, the larger angle being associated with the intramolecular hydrogen bond. The molecules are connected by intermolecular carboxylic acid O—H···O hydrogen bonds (Table 1) giving one-dimensional chains which extend along the *b* axial direction and give an overall two-dimensional network structure through π–π interactions between the quinoline rings [minimum ring centroid separation, 3.6068 (6) Å] (Fig. 2).

### Experimental

3-Nitropthalonitrile (1.73 g, 10.0 mmol), 8-hydroxyquinoline (1.45 g, 10.0 mmol) and K_2_CO_3_ (4.14 g, 30.0 mmol) were suspended in dry DMF (20 ml) and stirred at room temperature under a nitrogen atmosphere for 4 h. The reaction mixture was then poured into water (200 ml), and the crude product was separated by filtration and purified by column chromatography on silica gel using CH_2_Cl_2_ as an eluent. After removal of the solvent by rotary evaporation, 2.25 g of 3-(quinolin-8-yloxy)-phthalonitrile was obtained in a yield of 83%. Under nitrogen, 2.71 g, 10.0 mmol) of this compound and KOH (1.20 g, 30.0 mmol) were suspended in 30 ml of distilled water and refluxed until the solution turned clear. After being cooled to room temperature, the pH of the reaction mixture was slowly adjusted to about 5–6 using HCl (6.0 mol/*L*) with stirring. The solid product was separated by filtration, and then washed successively with water (3 times 30 ml). After drying under vacuum, 2.78 g of final produc was obtained in a yield of 91%. The solid was dissolved in methyl alcohol and the filtered solution was evaporated slowly at room temperature for 5–10 days, giving colorless crystals suitable for X-ray structure analysis.

### Refinement

Carboxylic acid H atoms were located in a difference-Fourier analysis and their positional and isotropic displacement parameters were refined. Other H-atoms were placed in geometrically determined positions and were treated as riding, with C—H = 0.93 Å and with *U*_iso_(H) = 1.2*U*_eq_(C).

### Crystal data

**Table:**

C_17_H_11_NO_5_	*F*(000) = 640
*M**_r_* = 309.27	*D*_x_ = 1.520 Mg m^−^^3^
Monoclinic, *P*2_1_/*c*	Mo *K*α radiation, λ = 0.71073 Å
Hall symbol: -P 2ybc	Cell parameters from 4908 reflections
*a* = 7.7337 (15) Å	θ = 3.2–27.6°
*b* = 11.580 (2) Å	µ = 0.11 mm^−^^1^
*c* = 15.260 (3) Å	*T* = 293 K
β = 98.43 (3)°	Plate, colourless
*V* = 1351.9 (5) Å^3^	0.47 × 0.45 × 0.10 mm
*Z* = 4	

### Data collection

**Table:**

Rigaku Saturn 724 CCD area-detector diffractometer	3079 independent reflections
Radiation source: fine-focus sealed tube	2899 reflections with *I* > 2σ(*I*)
Graphite monochromator	*R*_int_ = 0.041
ω scans	θ_max_ = 27.6°, θ_min_ = 3.2°
Absorption correction: numerical (*NUMABS*; Higashi, 2000).	*h* = −9→10
*T*_min_ = 0.948, *T*_max_ = 0.989	*k* = −14→14
11030 measured reflections	*l* = −19→19

### Refinement

**Table:**

Refinement on *F*^2^	Primary atom site location: structure-invariant direct methods
Least-squares matrix: full	Secondary atom site location: difference Fourier map
*R*[*F*^2^ > 2σ(*F*^2^)] = 0.058	Hydrogen site location: inferred from neighbouring sites
*wR*(*F*^2^) = 0.157	H atoms treated by a mixture of independent and constrained refinement
*S* = 1.27	*w* = 1/[σ^2^(*F*_o_^2^) + (0.0659*P*)^2^ + 0.6417*P*] where *P* = (*F*_o_^2^ + 2*F*_c_^2^)/3
3079 reflections	(Δ/σ)_max_ < 0.001
216 parameters	Δρ_max_ = 0.30 e Å^−^^3^
1 restraint	Δρ_min_ = −0.29 e Å^−^^3^

### Special details

**Table:**

Geometry. All esds (except the esd in the dihedral angle between two l.s. planes) are estimated using the full covariance matrix. The cell esds are taken into account individually in the estimation of esds in distances, angles and torsion angles; correlations between esds in cell parameters are only used when they are defined by crystal symmetry. An approximate (isotropic) treatment of cell esds is used for estimating esds involving l.s. planes.
Refinement. Refinement of F^2^ against ALL reflections. The weighted R-factor wR and goodness of fit S are based on F^2^, conventional R-factors R are based on F, with F set to zero for negative F^2^. The threshold expression of F^2^ > 2sigma(F^2^) is used only for calculating R-factors(gt) etc. and is not relevant to the choice of reflections for refinement. R-factors based on F^2^ are statistically about twice as large as those based on F, and R- factors based on ALL data will be even larger.

### Fractional atomic coordinates and isotropic or equivalent isotropic displacement parameters (Å^2^)

**Table:**

	*x*	*y*	*z*	*U*_iso_*/*U*_eq_	
O4	0.50110 (19)	0.69917 (13)	0.20095 (9)	0.0243 (3)	
O1	0.25866 (17)	0.79156 (12)	−0.00275 (9)	0.0188 (3)	
O5	0.2260 (2)	0.76160 (13)	0.18306 (9)	0.0256 (4)	
N1	−0.0436 (2)	0.81385 (14)	0.07072 (11)	0.0193 (4)	
O3	0.3439 (2)	0.47845 (13)	0.24357 (10)	0.0323 (4)	
O2	0.4250 (2)	0.33268 (13)	0.16305 (10)	0.0299 (4)	
C1	−0.3499 (3)	0.89691 (17)	−0.02677 (15)	0.0238 (4)	
H1	−0.4540	0.9225	−0.0593	0.029*	
C2	−0.3460 (3)	0.86376 (18)	0.05908 (15)	0.0247 (4)	
H2	−0.4464	0.8680	0.0859	0.030*	
C3	−0.1888 (3)	0.82314 (17)	0.10662 (14)	0.0225 (4)	
H3	−0.1864	0.8019	0.1656	0.027*	
C4	−0.1965 (3)	0.89267 (17)	−0.06673 (14)	0.0210 (4)	
C5	−0.0436 (2)	0.84770 (16)	−0.01521 (12)	0.0182 (4)	
C6	−0.1882 (3)	0.93173 (18)	−0.15387 (14)	0.0253 (5)	
H6	−0.2878	0.9605	−0.1886	0.030*	
C7	−0.0340 (3)	0.92718 (19)	−0.18686 (14)	0.0259 (5)	
H7	−0.0286	0.9559	−0.2433	0.031*	
C8	0.1181 (3)	0.87963 (18)	−0.13695 (13)	0.0221 (4)	
H8	0.2218	0.8761	−0.1609	0.027*	
C9	0.1119 (2)	0.83900 (16)	−0.05353 (13)	0.0188 (4)	
C10	0.2595 (2)	0.67061 (16)	−0.00022 (13)	0.0179 (4)	
C11	0.2268 (3)	0.60626 (19)	−0.07763 (13)	0.0252 (5)	
H11	0.1961	0.6424	−0.1321	0.030*	
C12	0.2408 (3)	0.4877 (2)	−0.07202 (14)	0.0303 (5)	
H12	0.2187	0.4433	−0.1232	0.036*	
C13	0.2875 (3)	0.43417 (18)	0.00914 (14)	0.0262 (5)	
H13	0.2971	0.3542	0.0120	0.031*	
C14	0.3200 (3)	0.49906 (17)	0.08628 (13)	0.0194 (4)	
C15	0.3055 (2)	0.61968 (17)	0.08224 (12)	0.0172 (4)	
C16	0.3644 (3)	0.43696 (17)	0.17291 (13)	0.0197 (4)	
C17	0.3492 (3)	0.69741 (16)	0.16191 (12)	0.0191 (4)	
H2A	0.447 (5)	0.295 (4)	0.218 (3)	0.085 (13)*	
H1A	0.052 (4)	0.794 (4)	0.110 (2)	0.102 (15)*	

### Atomic displacement parameters (Å^2^)

**Table:**

	*U*^11^	*U*^22^	*U*^33^	*U*^12^	*U*^13^	*U*^23^
O4	0.0257 (8)	0.0249 (8)	0.0204 (7)	−0.0003 (6)	−0.0027 (6)	−0.0041 (6)
O1	0.0191 (7)	0.0175 (7)	0.0193 (7)	0.0007 (5)	0.0012 (5)	0.0013 (5)
O5	0.0315 (8)	0.0255 (8)	0.0193 (7)	0.0092 (6)	0.0017 (6)	−0.0038 (6)
N1	0.0204 (8)	0.0174 (8)	0.0201 (8)	−0.0001 (6)	0.0026 (6)	−0.0020 (6)
O3	0.0535 (11)	0.0249 (8)	0.0204 (8)	0.0080 (7)	0.0113 (7)	0.0022 (6)
O2	0.0490 (10)	0.0205 (8)	0.0193 (8)	0.0094 (7)	0.0020 (7)	0.0021 (6)
C1	0.0200 (9)	0.0159 (9)	0.0342 (12)	0.0013 (7)	−0.0005 (8)	−0.0048 (8)
C2	0.0204 (10)	0.0214 (10)	0.0329 (11)	−0.0009 (8)	0.0063 (8)	−0.0050 (8)
C3	0.0259 (10)	0.0188 (10)	0.0236 (10)	−0.0015 (8)	0.0063 (8)	−0.0044 (8)
C4	0.0198 (9)	0.0151 (9)	0.0268 (10)	−0.0004 (7)	−0.0011 (8)	−0.0015 (7)
C5	0.0195 (9)	0.0150 (9)	0.0194 (9)	−0.0010 (7)	0.0004 (7)	−0.0020 (7)
C6	0.0259 (11)	0.0196 (10)	0.0278 (11)	0.0001 (8)	−0.0045 (8)	0.0030 (8)
C7	0.0306 (11)	0.0252 (11)	0.0204 (10)	−0.0024 (9)	−0.0014 (8)	0.0057 (8)
C8	0.0243 (10)	0.0201 (10)	0.0219 (10)	−0.0021 (8)	0.0033 (8)	0.0021 (7)
C9	0.0200 (9)	0.0164 (9)	0.0191 (9)	0.0006 (7)	−0.0001 (7)	−0.0003 (7)
C10	0.0176 (9)	0.0173 (9)	0.0191 (9)	0.0017 (7)	0.0031 (7)	−0.0001 (7)
C11	0.0313 (11)	0.0267 (11)	0.0160 (9)	0.0061 (9)	−0.0016 (8)	−0.0002 (8)
C12	0.0451 (13)	0.0255 (11)	0.0179 (10)	0.0053 (10)	−0.0039 (9)	−0.0066 (8)
C13	0.0357 (12)	0.0180 (10)	0.0226 (10)	0.0024 (8)	−0.0031 (9)	−0.0036 (8)
C14	0.0206 (9)	0.0187 (10)	0.0183 (9)	0.0015 (7)	0.0010 (7)	−0.0008 (7)
C15	0.0161 (8)	0.0178 (9)	0.0177 (9)	0.0003 (7)	0.0025 (7)	−0.0012 (7)
C16	0.0221 (9)	0.0166 (9)	0.0201 (10)	−0.0022 (7)	0.0019 (7)	−0.0001 (7)
C17	0.0257 (10)	0.0158 (9)	0.0154 (9)	−0.0011 (7)	0.0024 (7)	0.0011 (7)

### Geometric parameters (Å, º)

**Table:**

O4—C17	1.237 (2)	C5—C9	1.416 (3)
O1—C9	1.390 (2)	C6—C7	1.361 (3)
O1—C10	1.401 (2)	C6—H6	0.9300
O5—C17	1.286 (2)	C7—C8	1.416 (3)
N1—C3	1.324 (3)	C7—H7	0.9300
N1—C5	1.369 (3)	C8—C9	1.365 (3)
N1—H1A	0.904 (19)	C8—H8	0.9300
O3—C16	1.212 (2)	C10—C15	1.388 (3)
O2—C16	1.312 (2)	C10—C11	1.388 (3)
O2—H2A	0.93 (4)	C11—C12	1.379 (3)
C1—C2	1.361 (3)	C11—H11	0.9300
C1—C4	1.412 (3)	C12—C13	1.385 (3)
C1—H1	0.9300	C12—H12	0.9300
C2—C3	1.403 (3)	C13—C14	1.388 (3)
C2—H2	0.9300	C13—H13	0.9300
C3—H3	0.9300	C14—C15	1.402 (3)
C4—C6	1.415 (3)	C14—C16	1.500 (3)
C4—C5	1.420 (3)	C15—C17	1.511 (3)
			
C9—O1—C10	114.24 (14)	C7—C8—H8	120.1
C3—N1—C5	119.49 (18)	C8—C9—O1	121.25 (18)
C3—N1—H1A	114 (3)	C8—C9—C5	120.59 (18)
C5—N1—H1A	126 (3)	O1—C9—C5	118.12 (17)
C16—O2—H2A	110 (3)	C15—C10—C11	122.27 (18)
C2—C1—C4	120.31 (19)	C15—C10—O1	116.70 (16)
C2—C1—H1	119.8	C11—C10—O1	120.90 (17)
C4—C1—H1	119.8	C12—C11—C10	118.60 (19)
C1—C2—C3	119.16 (19)	C12—C11—H11	120.7
C1—C2—H2	120.4	C10—C11—H11	120.7
C3—C2—H2	120.4	C11—C12—C13	120.61 (19)
N1—C3—C2	122.5 (2)	C11—C12—H12	119.7
N1—C3—H3	118.8	C13—C12—H12	119.7
C2—C3—H3	118.8	C12—C13—C14	120.5 (2)
C1—C4—C6	123.50 (19)	C12—C13—H13	119.8
C1—C4—C5	117.26 (19)	C14—C13—H13	119.8
C6—C4—C5	119.23 (19)	C13—C14—C15	119.92 (18)
N1—C5—C9	119.65 (17)	C13—C14—C16	118.52 (18)
N1—C5—C4	121.24 (18)	C15—C14—C16	121.53 (17)
C9—C5—C4	119.11 (18)	C10—C15—C14	118.12 (17)
C7—C6—C4	119.90 (19)	C10—C15—C17	118.28 (17)
C7—C6—H6	120.1	C14—C15—C17	123.46 (17)
C4—C6—H6	120.1	O3—C16—O2	124.23 (19)
C6—C7—C8	121.31 (19)	O3—C16—C14	123.45 (18)
C6—C7—H7	119.3	O2—C16—C14	112.32 (17)
C8—C7—H7	119.3	O4—C17—O5	123.76 (18)
C9—C8—C7	119.73 (19)	O4—C17—C15	118.82 (17)
C9—C8—H8	120.1	O5—C17—C15	117.37 (17)
			
C4—C1—C2—C3	−1.3 (3)	C9—O1—C10—C11	49.8 (2)
C5—N1—C3—C2	1.8 (3)	C15—C10—C11—C12	−0.1 (3)
C1—C2—C3—N1	−1.1 (3)	O1—C10—C11—C12	175.68 (19)
C2—C1—C4—C6	−176.32 (19)	C10—C11—C12—C13	−0.3 (4)
C2—C1—C4—C5	3.0 (3)	C11—C12—C13—C14	0.3 (4)
C3—N1—C5—C9	179.36 (18)	C12—C13—C14—C15	0.0 (3)
C3—N1—C5—C4	0.0 (3)	C12—C13—C14—C16	178.0 (2)
C1—C4—C5—N1	−2.3 (3)	C11—C10—C15—C14	0.4 (3)
C6—C4—C5—N1	176.99 (18)	O1—C10—C15—C14	−175.46 (16)
C1—C4—C5—C9	178.31 (17)	C11—C10—C15—C17	176.33 (18)
C6—C4—C5—C9	−2.4 (3)	O1—C10—C15—C17	0.4 (3)
C1—C4—C6—C7	178.45 (19)	C13—C14—C15—C10	−0.4 (3)
C5—C4—C6—C7	−0.8 (3)	C16—C14—C15—C10	−178.36 (17)
C4—C6—C7—C8	2.6 (3)	C13—C14—C15—C17	−176.09 (19)
C6—C7—C8—C9	−1.0 (3)	C16—C14—C15—C17	6.0 (3)
C7—C8—C9—O1	179.88 (18)	C13—C14—C16—O3	−157.0 (2)
C7—C8—C9—C5	−2.3 (3)	C15—C14—C16—O3	20.9 (3)
C10—O1—C9—C8	−101.6 (2)	C13—C14—C16—O2	22.0 (3)
C10—O1—C9—C5	80.5 (2)	C15—C14—C16—O2	−160.01 (18)
N1—C5—C9—C8	−175.44 (18)	C10—C15—C17—O4	−112.9 (2)
C4—C5—C9—C8	3.9 (3)	C14—C15—C17—O4	62.7 (3)
N1—C5—C9—O1	2.5 (3)	C10—C15—C17—O5	64.5 (2)
C4—C5—C9—O1	−178.14 (16)	C14—C15—C17—O5	−119.8 (2)
C9—O1—C10—C15	−134.25 (17)		

### Hydrogen-bond geometry (Å, º)

**Table:**

*D*—H···*A*	*D*—H	H···*A*	*D*···*A*	*D*—H···*A*
O2—H2*A*···O4^i^	0.93 (4)	1.67 (4)	2.584 (2)	165 (4)
N1—H1*A*···O5	0.90 (2)	1.67 (2)	2.570 (2)	179 (5)

Symmetry code: (i) −*x*+1, *y*−1/2, −*z*+1/2.

## References

[bb1] Etter, M. C., MacDonald, J. C. & Bernstein, J. (1990). *Acta Cryst.* B**46**, 256–262. 10.1107/s0108768189012929 2344397

[bb2] Higashi, T. (2000). *NUMABS* Rigaku Corporation, Tokyo, Japan.

[bb3] Rigaku (2007). *CrystalClear* Rigaku Corporation, Tokyo, Japan.

[bb4] Sheldrick, G. M. (2008). *Acta Cryst.* A**64**, 112–122. 10.1107/S0108767307043930 18156677

[bb5] Spek, A. L. (2009). *Acta Cryst.* D**65**, 148–155. 10.1107/S090744490804362X PMC2631630 19171970

[bb6] Su, Y., Zang, S., Li, Y., Zhu, H. & Meng, Q. (2007). *Cryst. Growth Des.* **7**, 1277–1283.

[bb7] Wang, H., Zhang, D., Sun, D., Chen, Y., Zhang, L.-F., Tian, L., Jiang, J. & Ni, Z.-H. (2009). *Cryst. Growth Des.* **9**, 5273–5282.

[bb8] Zang, S., Su, Y., Li, Y., Zhu, H. & Meng, Q. (2006). *Inorg. Chem. Commun.* **9**, 337–340.

[bb9] Zang, S., Su, Y., Song, Y., Li, Y., Ni, Y., Zhu, H. & Meng, Q. (2006). *Cryst. Growth Des.* **6**, 2369–2375.

